# Recurrent/Metastatic Nasopharyngeal Carcinoma Treatment from Present to Future: Where Are We and Where Are We Heading?

**DOI:** 10.1007/s11864-023-01101-3

**Published:** 2023-06-15

**Authors:** Juan Jose Juarez-Vignon Whaley, Michelle Afkhami, Mykola Onyshchenko, Erminia Massarelli, Sagus Sampath, Arya Amini, Diana Bell, Victoria M. Villaflor

**Affiliations:** 1 Health Science Research Center, Faculty of Health Science, Universidad Anahuac Mexico, State of Mexico, Naucalpan de Juárez, Mexico; 2 Department of Pathology, City of Hope Comprehensive Cancer Center, Duarte, CA USA; 3 Department of Medical Oncology, City of Hope Comprehensive Cancer Center, Duarte, 1500 East Duarte Road. , Duarte, CA 91010 USA; 4 Department of Radiation Oncology, City of Hope Comprehensive Cancer Center Duarte, Duarte, CA USA

**Keywords:** Nasopharyngeal carcinoma, Recurrent/metastatic disease, Management NPC, Epstein-Barr virus positivity, Targeted therapy, Immunotherapy, Clinical trials

## Abstract

Nasopharyngeal carcinoma (NPC) is distinct in its anatomic location and biology from other epithelial head and neck cancer (HNC). There are 3 WHO subtypes, which considers the presence of Epstein-Barr virus (EBV) and other histopathology features. Despite the survival benefit obtained from modern treatment modalities and techniques specifically in the local and locally advanced setting, a number of patients with this disease will recur and subsequently die of distant metastasis, locoregional relapse, or both. In the recurrent setting, the ideal therapy approach continues to be a topic of discussion and current recommendations are platinum-based combination chemotherapy. Phase III clinical trials which led to the approval of pembrolizumab or nivolumab for head and neck squamous cell carcinoma (HNSCC) specifically excluded NPC. No immune checkpoint inhibitor therapy, to date, has been approved by the FDA to treat NPC although the National Comprehensive Cancer Network (NCCN) recommendations do include use of these agents. Hence, this remains the major challenge for treatment options. Nasopharyngeal carcinoma is challenging as it is really 3 different diseases, and much research is required to determine best options and sequencing of those options. This article is going to address the data to date and discuss ongoing research in EBV + and EBV – inoperable recurrent/metastatic NPC patients.

## Introduction

Nasopharyngeal carcinoma (NPC) arises in the nasopharynx and differs from other head and neck cancer (HNC) in histology, epidemiology, molecular pathogenesis, and treatment response. In 2020, there were 133,354 new cases and 80,008 deaths in both sexes and all ages [1]. Its etiology involves factors, such as genetic predisposition, Epstein-Barr virus (EBV) infection, and environmental risk factors (smoking, salt-cured food). It is a rare malignant epithelial tumor endemic to Southern and Southeast China. Histologically, NPC is categorized by The World Health Organization into three types. Keratinizing squamous cell carcinoma (type 1) consists of well-differentiated cells that produce keratin. Nonkeratinizing carcinoma, which can be differentiated (type 2) or undifferentiated (type 3), these do not produce keratin. Basaloid squamous cell carcinoma (subset of type 3) is also nonkeratinizing and characterized by less differentiated cell types and is associated with aggressive disease [2]. Subtypes II and III are strongly related to EBV infection, the main risk factor for NPC and subtype III is the most predominant histological subtype in Asia, whereas, in non-endemic regions, NPC type 1 is the most frequent histological subtype [3, 4].

EBV driven NPC is of particular interest in that up to 20% of all cancers may be related to a bacterial or viral infection [5]. A persistent EBV viral infection may not produce symptoms; however, specific EBV proteins such as EBV nuclear antigen 1 (EBNA1), latent membrane proteins (LMPs), and the BamHI-A fragment of the EBV genome have been identified in NPC cells mediating tumorigenesis and avoiding the immune system [6, 7]. EBV is located in the cancer cells of nearly every patient with advanced stage EBV driven NPC and contributes in disease development and progression [5].

Unfortunately, diagnosis is generally locally advanced or metastatic with recurrency and distant metastasis being the most common cause of death in patients. Survival has improved for patients with locoregionally advanced disease with successful chemoradiation strategies over the past decades. Despite these advances, 30% still experience recurrence/metastatic (R/M) disease [5]. For these patients, median overall survival (OS) ranges between 10 and 36 months and those in stage IV, the 5-year survival rate is less than 40% even in high-income countries [8, 9]. Treatment generally includes platinum-based chemotherapy and in selected patients targeted and immunotherapies, which are ongoing areas of research [10, 11].

The present review offers an understanding in the management of R/M NPC patients with focus in the ongoing and investigational agents to improve patient survival.

## Tumor biology

### Molecular and immunohistochemistry

Advances in new technologies in both the molecular genomics and immunohistochemistry (IHC) have allowed understanding cancer cells interaction, facilitate diagnosis, prognosis, and created new therapeutic modalities. In this section, we offer an understanding of these techniques’ utilities in the tumor biology of NPC.

#### Next generation sequencing

Genomic alterations including mutations, copy number variation (CNV), and fusions play an important role in driving tumorigenesis detected by next generation sequencing (NGS). NPC has genomic instabilities with a wide variety of somatic mutations and more frequent CNV than other head and neck cancers. EBV+ NPC WHO 2 and 3 is a homogenous cancer driven largely by NF-kB signaling caused by somatic aberrations of negative regulators including *LMP1, CYLD, TRAF*, and *NFKBIA* or overexpression of EBV oncoproteins [12, 13]. These negative regulator gene mutations affect the NF-kB pathway, cell cycle, cell death, EBV infection, and carcinogenesis.

Per COSMIC somatic mutations database, the most common somatic mutations have been identified in genes including *TP53, KMT2C, NOTCH2, BRCA1* and *2, PTCH1, IL7R, KDR, EGFR,* and *PIK3CA* [14]. Somatic mutations of *TP53* have been identified as a high-rate mutation in NPC [15]. Function genes are enriched in multiple signaling pathways during the development of NPC which include *RASSF1*, *PIK3CA*, *MAD1L1*, HLA-A/B/C, *LTBR*, *CCDND1*, *NFKBIA*, *CYLD*, and *TP53* [16]. These genes alter the NF-kB pathway and are key drivers for malignant transformation of nasopharyngeal cells. *PI3K/AKT/mTOR* pathway mutations have been identified promoting proliferation, migration, and inhibition of apoptosis [16]. Mutations in the EBV genome, *LMP1* gene enhance proliferation, migration, and NF-kB activation [17]. *KMT2C/2C, EP300, KDM5A* and *BamH1-A* mutations, function, and importance are yet to be defined [12, 18].

Studies in Asian and Southeastern European population have demonstrated *BRCA1* as a common mutated gene and unfavorable prognosis followed by *BRCA2, TP53*, and *KRAS*, while EBV positivity was notes as a favorable prognosis factor [19]. Dysplastic nasopharynx cells have identified carcinogenic genes (*CDKN2A, RASSF1A, TGFBR2)* with acquired lesions in chromosomes 3p, 9p, 11q, 12, 13q, 14q, and 16q [13]. Genetic alterations in *hMLH1* by EBV infection, inactivation of *PMS2*, and negative regulators of the NK-kB pathway like *TRAF3, CYLD, NFKBIA, LMP1*, and *NLRC5* have been identified in non-keratinizing NPC EBV+ studies [20]. Information regarding germline mutations for NPC please refer to our locally advanced manuscript.

There are various pathways that repair DNA double-strand breaks in healthy cells, and mutations in genes encoding homologous recombination proteins are associated in the development of malignancies [21–23]. DNA repair defects vary depending on the cancer type, for example BRCA gene mutations predispose patients to breast cancer, ovarian, pancreatic, prostate, and non-small cell carcinoma (NSCLC) [24]. Interestingly, BRCA genomic mutations have been found in NPC. In a cohort of European NPC patients, the most common somatic mutation was BRCA1 (54%) followed by BRCA2 (29%) [19]. Additionally, BRCA1 mutations were associated with an unfavorable prognosis. The identification of BRCA1 may serve as a target for PARP inhibitors, and may be beneficial overcoming defects in DNA repair and enhancing chemo or radiation effects [24, 25].

PARP has been implicated in playing a role in the EBV lytic cycle [26, 27]. Hence, PARP1 inhibition may be an effective treatment for NPC-EBV+ tumors and EBV associated malignancies. Combination treatment with immunotherapy and PARP inhibitors is a promising therapy for NPC as these tumor may generate more neoantigens, elevate tumor immunogenicity, and improve immune response. The POINT trial (NCT04825990) among other trials evaluating ICIs + PARP inhibitors are under investigator and will be further mentioned in the manuscript.

#### Somatostatin receptor 2 by IHC

Even though there are multiple genes that play a role in the pathogenesis of NPC, cohorts in R/M NPC have demonstrated a high expression of somatostatin receptor 2 (SSTR2) by IHC. SSTR2, a G protein coupled cell surface receptor, inhibits cell proliferation, and is mainly expressed in neuroendocrine tumors [28]. Lechner et al. demonstrated the expression of SSTR2 in 252 of 311 NPC primary R/M samples localized in the plasma membrane with enriched SSTR2 expression in EBV + and non-keratinizing subtypes, and no difference in levels between recurrent and metastatic disease [9]. In this study, there is a suggestion that SSTR2 expression may work as a prognostic factor, with higher expression associated with increased rates of survival. The upregulation of SSTR2 in EBV + NPC, Lechner et al. identified an aberrant activation of the NF-kB signaling pathway via the EBV oncoprotein LMP1. SSTR2 overexpression was also demonstrated in a case series via octreotide PET/CT [29]. This principal was translated to NPC in which studies demonstrated an increased uptake of specific radiocontrast in EBV + NPC [30–32]. The discovery understanding the role of SSTR2 in NPC pathogenesis which its expression is induced by LMP1 via the NF-kB pathway has been of great importance. High expression of SSTR2 is helpful as a diagnostic biomarker via imaging. Interestingly, SSTR2 expression is also significantly expressed in pulmonary lymphoepithelioma-like carcinoma, also an EBV-associated cancer [33].

Therapeutic strategies targeting SSTR2 have also been studied with agonists demonstrating effective growth controlling effects in tumors with <10% proliferation rates [34]. This hypothesis was studied in NPC cells in which SSTR2 agonists (lanreotide, octreotide, and PEN-221) were evaluated as investigational treatment strategies Octreotide and lanreotide did not affect NPC tumor proliferation, different to what has been observed in more indolent neuroendocrine tumors; however, PEN-221 did demonstrate an increase in OS and anti-tumor efficacy. It is important to highlight that these treatment strategies are yet to prove clinical efficacy and are merely investigational at the moment These peptides can be chelated with cytotoxic nuclides and have been proven effective in neuroendocrine tumors and in one case of NPC [28, 35]. Further research is required regarding the use of SSTR2 agonists as therapeutic options for NPC.

#### Programmed cell death protein 1 and tumor infiltrating lymphocytes by IHC

The immunological environment of NPC is unique due to the heavy infiltration of CD3+,CD8+, T-regulatory cells, natural killer cells, neutrophils, and dendritic cells within the stroma, with these playing an important role in growth and tumor invasion [36]. Low density of CD8+, neutrophils, and mast cells are associated with a longer survival while high density natural killer cells improve survival [36]. Additionally, NPC cells similar to many cancers have well-established methods of immune system evasion, mainly via de programmed cell death protein 1 (PD-1)/PD-L1 axis. NPC overexpress PD-L1 in 50–80% of tumors, especially in EBV+ due to the effects that LMP1 have in regulating PD-L1 expression [37, 38]. This overexpression may be associated with better efficacy to immune checkpoint inhibitors (ICI), as this principle has been demonstrated in other solid tumors such as NSCLC. The manipulation of the PD-1/PD-L1 axis via ICIs induces an antitumor response. Another reason why ICIs are being used in NPC is due to its high antigenicity, since it is an EBV driven cancer neoantigens such as LMP1, LMP2, and EBNA1 are overexpressed [39, 40].

ICIs have become the focus of intense research in recent years as one of the main therapeutic strategies for NPC. ICIs are effective at helping antitumor immunity [41]. PD-1 has a major impact adjusting the function of lymphocytes and managing the immune-system, making it one of the most comprehensively researched regulators [41]. The relation between PD-1 and PD-L1 can avoid T cells from activation and proliferation, preceding to tumor recurrence and metastasis. Zhang et al. studied the link between PD-1/PD-L1 expression and posttreatment results in NPC patients and found increased levels of PD-L1 expression in the malignant tissues of NPC patients [42]. When assessing PD-L1 expression in EBV- associated NPC patients, 16/18 (89%) of subjects showed positive PD-L1 staining in malignant cells [41]. This implies a link between PD-1 and NPC recurrence, metastasis, and progression. The measurement of PD-1 via immunohistochemistry at time of biopsy has the goal of predicting patients that would respond better to ICIs and therefore could be candidates for potential 1^st^ line combination therapies. These findings helped prove the efficacy of ICI (toripalimab) + gemcitabine-cisplatin chemotherapy as first-line treatment in the JUPITER-2 study.

## Treatment

### Chemotherapy

#### Metastatic and recurrent disease

Patients (15–58%) will experience inoperable/recurrent or metastatic disease and will require systemic therapy [43]. Standard chemotherapy includes a platinum doublet chemotherapy. However, one must encourage patients to enroll in clinical trials as the gained knowledge may improve patient survival and alter standard of care.

NPC sensitive to platinum-based regimen, preferably cisplatin. These effects have been demonstrated in various R/M and locally advanced trials. In R/M disease, the combination of paclitaxel, cisplatin, and 5-FU obtained an overall response rate (ORR) of 78.9%, disease control rate of 93.6%, OS of 24.8 months, and progression free survival (PFS) of 22.7 months [44]. One of the most important trials to establish cisplatin-gemcitabine as preferred regimen in EBV+ NPC done by Zhang et al. improved PFS by 1.4 months and OS by 3 months when compared to cisplatin-fluorouracil [45, 46]. This trial established maximum of 6 cycles as first-line treatment with an acceptable safety profile. Future research should identify factors which predict patient response. The NCT01365208 trial identified early PET-CT response (>50% drop in sum of SUVmax lesions) and a plasma EBV-DNA clearance ≤ 10 days, as predictors of patient survival and subsequent response to chemotherapy [47].

Platinum combination therapy with fluorouracil, carboplatin, taxanes, or cetuximab is a reasonable option for patient’s intolerant to cisplatin-gemcitabine. Regardless of the alternatives, cisplatin must be maintained when possible. One trial compared five different cisplatin-based regimens and demonstrated higher response rates in the cisplatin-gemcitabine and cisplatin-paclitaxel-5FU regimens but no significant difference between the five regimens in PFS or OS [48]. Cisplatin-fluorouracil and cisplatin-paclitaxel were the preferred alternatives. A phase III trial in advanced HNC demonstrated no significant difference in OS or ORR between therapies [49]. Carboplatin-paclitaxel proved to be an effective alternative with a PFS of 7 months, 12-month OS, 59% ORR, and tolerable toxicities [50, 51]. Another alternative is capecitabine which demonstrated to be an efficacious alternative as induction chemotherapy (paclitaxel-cisplatin-capecitabine) followed by maintenance capecitabine alone, improving PFS, ORR, and duration of response with a tolerable toxicity [52]. Nevertheless, the decision to use maintenance chemotherapy is not standardized, and more studies are needed. Another alternative is cetuximab-carboplatin, demonstrating an ORR of 11.7%, 233 days OS, and manageable toxicities [53]. The use of targeted therapies including immunotherapies and molecular therapy will be discussed in a subsequent section.

Single-agent chemotherapy, in the first-line setting, is limited to patients that do not tolerate a combined regimen, are of older age or have a decreased performance status. Agents include platinum, fluorouracil or capecitabine, taxanes, gemcitabine, anthracyclines, methotrexate, bleomycin, ifosfamide, vinorelbine, and irinotecan [54–59]. See Table 1 for acceptable chemotherapeutic regimens for R/M NPC.

**Table 1:** Chemotherapies used in the management of R/M NPC with their corresponding response rate and survival outcomes

Trial/author	Regimens studied	n	EBV status	ORR (%)	PFS	OS
Combined chemotherapy						
Hong, et al., 2021 [47]	Gemcitabine-cisplatin vs 5FU-cisplatin	362	Optional: GP: 8 EBV-, 13 EBV+. FP:1 EBV-, 6 EBV +.	N/A	7.6% vs 0% (5-year PFS)	22.1 vs 18.6 months
Zhang et al., 2016 [46]	Gemcitabine-cisplatin vs 5FU-cisplatin	362	Optional (not reported)	64% vs 42%	7.0 vs 5.6 months	29.1 vs 20.9 months
Chen et al., 2013 [45]	Cisplatin-paclitaxel-5FU	95	N/A	78.9%	8.6 months	22.7 months
Jin et al., 2012 [49]	Cisplatin-5FU vs paclitaxel-cisplatin vs gemcitabine-cisplatin vs paclitaxel-cisplatin-5FU vs bleomycin-cisplatin-5FU	822	EBV -: 212 EBV +: 576	60.2% vs 61.7% vs 71.1% vs 69.1% vs 74.0%	5.0 vs 5.5 vs 6.6 vs 5.5 vs 6.0 months	19.5 vs 21.0 vs 21.5 vs 19.0 vs 21.0
Tan et al., 1999 [52]	Paclitaxel-carboplatin	32	N/A	75%	7.0 months	12.0 months
Yeo et al., 1998 [51]	Paclitaxel-carboplatin	27	N/A	59%	5.9 months	13.9 months
Second line chemotherapy						
Chan et al., 2005 [54]	Carboplatin-cetuximab (second line)	60	N/A	11.7%	81 days	233 days
Chua et al., 2000 [61]	Ifosfamide-5FU-leucovorin (second line)	18	N/A	56%	6.5 months	51% [1-year survival]
Single-agent chemotherapy						
Ngeow et al., 2011 [58]	Docetaxel	30	N/A	36.7%	5.3 months	12.8 months
Ciuleanu et al., 2008 [56]	Capecitabine	23	N/A	48%	15 months	62% [1-year survival]
Poon et al., 2005 [57]	Irinotecan	28	N/A	14%	3.9 months	11.4 months
Chua et al.,2003 [55]	Capecitabine	17	N/A	23.5%	4.9 months	7.6 months
Foo et al., 2002 [60]	Gemcitabine: chemo-naïve vs previously treated	52	N/A	28% vs 48%	3.6 vs 5.1 months	7.2 vs 10.5 months
Au et al., 1998 [59]	Paclitaxel	24	N/A	21.7%	2.5 months	12 months
Maintenance Chemotherapy						
Liu et al., 2022 [53]	Maintenance capecitabine vs best supportive care	104	N/A	25% vs 11.5%	35.9 vs 8.2 months	N/A

*EBV*, Epstein-Barr virus; *ORR*, overall response rate; *PFS*, progression-free survival; *OS*, overall survival; *N/A*, not available or not reported; *GP*, gemcitabine-platinum; *FP*, 5FU-platinum

#### Oligometastatic disease

The ESTRO-ASTRO consensus defines oligometastatic disease as 1–5 metastatic lesions with a controlled primary tumor being optional, but all metastatic sites must be safely treatable [60]. Patients with oligometastatic disease and good performance status with partial response to chemotherapy should be offered radiation consolidation therapy. This recommendation was reported in various retrospective trials; one in 197 patients demonstrated that palliative radiotherapy (RT) after chemotherapy improved 2-year metastatic survival rate compared to chemotherapy alone or best supportive care (57.7% vs. 32.7% vs. 1.6%); another trial in 448 patients who received RT demonstrated an improved OS, cancer-specific survival, and 50% reduced risk mortality compared to those who did not receive radiotherapy [61, 62]. More recently, two retrospective studies demonstrated an increased 3-year OS rate in favor of RT, improved OS in de novo metastatic disease mainly observed in low-risk patients (score ≤ 102) based on a prognostic model taking in consideration LDH, number of metastatic lesions, liver metastasis, posttreatment EBV DNA levels, and response of metastases to chemotherapy [63, 64].

Final recommendation of consolidative RT was demonstrated in a phase 3 trial where patients assigned to the chemotherapy + RT had a 24-month OS of 76.4% versus a 54.5% in those treated with chemotherapy alone as well as improving PFS (12.4 vs. 6.7 months) [65]. It is important to mention that this trial used cisplatin-5FU chemotherapy. Future clinical trials should address this effect in cisplatin-gemcitabine. These highlight the importance of locoregional RT in oligometastatic NPC.

Ongoing phase 2/3 trials focusing on RT in oligometastatic disease include trials studying camrelizumab + stereotactic RT (NCT04944914), consolidative RT + camrelizumab (NCT05128201) + chemotherapy, RT + PD-1 inhibitors (NCT05290194), whole-target consolidation RT (NCT05431764), and RT + toripalimab + chemotherapy (NCT05385926).

### Targeted and immunotherapy

Understanding cancer-genetics and tumor biology including molecular and immunological microenvironment is crucial as new therapeutic strategies are explored. As mentioned in the “Tumor Biology” section, the role of PD-L1, SSTR2, BRCA, and the relationship between EBV and NF-kB pathway play important roles in NPC tumorigenesis. One important pathway is the NF-κB transcription factor, which is upregulated in EBV + NPC. There is evidence that the abnormalities of NF-κB and signaling pathways that regulate its action are tied to cancer growth, development, and resistance to therapies [66–69]. These biomarkers including immunological microenvironment, cancer-genetics, and molecular biology have created new methods of treating NPC.

#### Immune checkpoint inhibitors

The addition of immune checkpoint inhibitors (ICIs) as initial therapy is a promising approach. It is important to mention that ICIs are still under development, mainly available under clinical trials. The use of ICIs as first-line therapy is combined always with chemotherapy, preferentially cisplatin-gemcitabine. The addition of ICIs such as toripalimab (IgG4 antibody versus PD-1), camrelizumab (PD-1 inhibitor), or tislelizumab (PD-1 inhibitor) to cisplatin-gemcitabine has significantly improved PFS [70–72]. This combination has had such an impact that ESMO-EURACAN clinical guidelines highlight the importance of adding camrelizumab or toripalimab to platinum-gemcitabine improving PFS as first-line metastatic endemic NPC [70, 71, 73].

These combination therapies have been approved in China with FDA approval pending in the USA. Hence, clinical trials should be strongly encouraged. Toripalimab, camrelizumab, and tislelizumab are currently used in Asia. Toripalimab + cisplatin-gemcitabine as first line improved PFS compared to placebo + standard chemotherapy (11.7 vs. 8.0 months) as well as reducing risk of death by 40% [70]. Camrelizumab + cisplatin-gemcitabine also improved PFS compared to cisplatin-gemcitabine alone (9.7 vs. 6.9 months) [71]. Finally, tislelizumab + cisplatin-gemcitabine improved patients PFS (9.6 vs. 7.4 months) with similar toxicities [72]. This study also reports second PFS which is the time from randomization to objective disease progression on subsequent-second line treatment or death and demonstrated a benefit in favor of tislelizumab (not reached vs. 13.9 months). These three clinical trials included their respective ICIs as maintenance monotherapy. This raises awareness of the importance that maintenance ICI therapy may have in controlling disease, reducing recurrence, and improving OS. These studies have included the use of nivolumab and pembrolizumab in combination with cisplatin-gemcitabine by the National Comprehensive Cancer Network (NCCN) guidelines in the management of R/M NPC.

ICIs have been studied in patients who progress on initial platinum-based chemotherapy. In 2017, the KEYNOTE-028 trial reported the antitumor activity and safety profile of pembrolizumab in previously treated patients with platinum-based chemotherapy. Pembrolizumab obtained an ORR of 25.9% and a manageable safety profile [43]. More recently, the KEYNOTE-122 trial showed no difference in OS between pembrolizumab (17.2 months) and chemotherapy (15.3 months), PFS (4.1 vs. 5.5 months) nor ORR (21.4 vs. 23.3%). Adverse events are less common with ICIs compared to chemotherapy [74]. Nivolumab, another option for chemo-refractory scenarios, demonstrated (NCI-9742) to have similar ORR as pembrolizumab (20.5%), disease control rate of 54.5%, OS of 17.1 months, and PFS of 2.8 months [75]. Similar survival and response benefits were observed in the CheckMate-358 trial, where nivolumab reported an ORR of 20.8%, disease control rate of 45.8%, and PFS of 2.4 months [76]. The NCI-9742 trial also correlated expression of PD-L1, HLA-A, HLA-B, and plasma clearance of EBV-DNA virus with ORR and survival, concluding that the loss of expression from one or both HLA-1 proteins was associated with a better PFS. Identifying HLA-A and HLA-B as prognostic factors. Lastly toripalimab in the POLARIS-02 trial reported an ORR of 21%, PFS of 2 months, and OS of 17 months [77]. It also reported that patients with a ≥ 50% decrease in EBV copy number had an improved ORR and those with genomic amplification in either the *11q13* region or *ETV6* genomic alterations responded poorer to toliparimab.

Other ICIs include camrelizumab (PD-1 inhibitor), as combined initial therapy with cisplatin-gemcitabine. One trial reported its use as monotherapy following 1^st^ line chemotherapy with a 31% ORR, PFS of 5.6 months and tolerable toxicities [78]. Furthermore, the CAPTAIN study, analyzing camrelizumab in chemo-refractory R/M NPC, reported an ORR of 28.2%, PFS of 3.7 months, and an OS of 17.4 months [79]. They identified that patients who express high stromal MHC-II cell density and PD-L1 result in better patient response. A publication by the American Association of Cancer Research analyzed the effects of spartalizumab (PD-1 inhibitor) in chemo-refractory patients. Even though there was no benefit in PFS or ORR, spartalizumab reported fewer grades 3–4 adverse events and long-lasting tumor responses especially in those with *IFN-y* signature, *TIM3,* and *LAG3* gene expression [80]. These clinical trials not only have the goal of discovering new drugs but also identifying prognostic biomarkers to help guide and identify patients that will benefit of these treatments.

ICIs have completely changed the management of R/M NPC patients. There is still need of evaluation of other checkpoint inhibitors and predicting efficacy in randomized trials.

#### Molecular therapy

Molecular targeted therapy does not appear to have useful clinical and survival advantages in R/M NPC with PARP inhibitors lately demonstrating a potential benefit. There are a few limitations associated with the studies such as small sample sizes, lack of phase III trials, and short duration of follow-up [66, 67, 69]. The current role of targeted therapy is limited and exploring these new therapies is because patients who relapse with distant metastasis tend to have a poor prognosis with median survival ranging from 5 to 11 months [81, 82].

##### PARP inhibitors

Inhibition of PARP1 protein is a novel target mechanism for treating NPC due to its relationship with BRCA mutation, alterations in different homologous-recombination genes, and PARP1 upregulation in NPC cells [83]. Cancer cells including NPC cells have mechanisms to avoid the immune system such as creating an immunosuppressive microenvironment and negative-costimulatory signals (PD-L1). Therefore, the PARP inhibitors + PD-1/PD-L1 inhibitors could enhance their anti-cancerous activities and be an effective treatment.

Most of the trials studying PARP inhibitors are ongoing; however, one study demonstrated olaparib to have apoptotic, DNA damage and cell-cycle arrest effects in NPC cells, and enhancing activity of chemo and radiotherapy [83]. In other studies, NFBD1 (nuclear protein) depletion enhances the effects of olaparib making it a strategy in those resistant to PARP inhibitors [84]. There are three main ongoing phase 2 clinical trials evaluating PD-1 + PARP inhibitors in R/M NPC who failed first-line chemotherapy. These trials include pembrolizumab (PD-1 inhibitor) + olaparib (NCT04825990), camrelizumab (PD-1 inhibitor) + fluzoparib (PARP inhibitor) (NCT04978012), and niraparib (PARP inhibitor) + sintilimab (PD-1 inhibitor) (NCT05162872), with their primary outcome being ORR.

##### EGFR inhibitors

Epidermal growth factor receptor (EGFR) has become a therapeutic target in multiple cancers including NSCLC, leukemias, and HNC with its expression associated with aggressive tumor phenotype; therefore, targeting it has become a treatment option in certain scenarios [85]. EGFR expression has been reported in up to 85% of NPC and its expression associated with poorer outcomes [86, 87]. Trials have studied the efficacy of EGFR inhibitors mainly in non-responders to first-line chemotherapy.

Chan et al. combined cetuximab with carboplatin demonstrating clinical activity with an ORR of 11.7%, 81 days of PFS, 233 days of OS, and only 31.7% of reported toxicities [53]. Cetuximab-carboplatin concluded to be an acceptable combination in platinum resistant R/M NPC. Additional EGFR tyrosine kinase inhibitors have also been evaluated. Gefitinib demonstrated clinical response in R/M HNSCC (ORR 10.4% and disease control rate 53%) [88]. Later, gefitinib demonstrated a safe delivery with no grades 3–4 toxicities reported; however, no patient obtained an ORR, PFS of 4 months, and OS of 16 months [89]. Another phase 2 trial also evaluated gefitinib in metastatic and locoregionally recurrent NPC. Results were similar with gefitinib being well tolerated but no ORR, a 2.7-month PFS, and 12-month OS [90]. More recently, erlotinib (EGFR tyrosine kinase inhibitor) in R/M NPC as maintenance therapy post-6 weeks of cisplatin-gemcitabine was well tolerated but no impact was observed in patients ORR or survival (6.9-month PFS; 12-month OS of 80%) [11]. Even though minimal efficacies were reported, these last two trials identified plasma EBV-DNA as a potential biomarker for treatment response. There was a radiological progression of disease associated with rising levels of plasma EBV-DNA and patients who had longest duration of stable disease were associated with undetectable levels [11, 90].

The use of EGFR inhibitors has minimal effect in the management of R/M NPC and should only be considered for clinical trials in cases with recurrence/progression after first-line chemotherapy or chemo-resistant.

##### VEGFR inhibitors

Targeting the vascular endothelial growth factor receptor (VEGFR) is another molecular therapy that has been studied. VEGFR overexpression has been found in 60–67% of NPCs and is correlated with poor survival [91, 92]. The VEGFR pathway plays an important role in angiogenesis, tumor growth and metastasis, thus its inhibition is a potential target [93, 94].

Sorafenib is a multi-kinase inhibitor that blocks the serine/threonine kinases C-Raf and B-Raf, VEGFR-2 & 3 and platelet-derived-growth factor receptor and has been studied in two trials. In 2007, sorafenib was evaluated as a single agent in R/M HNSCC and NPC, with modest efficacy, an ORR of 3.7%, PFS of 1.8 months, and OS of 4.2 months [95]. However, the drug was well tolerated with no grade 4 toxicities and demonstrated an anti-cancerous effect with a decrease expression of pERK, Ki67, and Mcl-1 (antiapoptotic protein) posttreatment. Following these results, a phase 2 trial evaluated sorafenib + cisplatin-5FU as first-line therapy, reporting an ORR of 77.8%, PFS of 7.2 months and OS of 11.8 months, but with a 22% incidence of hemorrhage [96]. Making sorafenib combined with chemotherapy a feasible therapy in R/M NPC patients with the importance of standardizing the dose to avoid adverse events. Pazopanib (multi-kinase inhibitor) was studied in endemic cases of WHO II and III R/M NPC who had failed to ≥ 1 chemotherapy lines. Clinical benefit rate (CBR) was 54.5%, with an ORR of 6.1% (only partial response), PFS of 4.4 months, and OS of 10.8 month [97]. Importantly, one death was reported due to epistaxis, 78.6% reported grades 3–4, and a significant reduction in tumor blood flow. Even though pazopanib proved anti-cancerous effects, its use with additional cytotoxic drugs may limit its administration due to potentially deathly adverse events. Hui et al. analyzed the efficacy of sunitinib and demonstrated a high incidence of hemorrhage (64%) mainly from the upper aerodigestive tract in patients with prior high-dose RT and sunitinib having minimal clinical activity [98]. The hemorrhagic events made the researchers include a safety precaution to exclude patients who received previous RT and/or the tumor invading major vascular structures from VEGFR inhibitors.

VEGFR inhibitors are still being studied. In 2018, axitinib was studied in previously treated patients excluding those with local recurrence or vascular invasion (due to increases risk of hemorrhagic events). The CBR was 78.4% at 3 months and 43.2% at 6 months, with a PFS of 5 months and OS of 10.4 months, with hemorrhages reported only as grades 1–2 and the most common adverse event being hypertension [99]. Hypertension which was identified as a biomarker for clinical efficacy and toxicity. Axitinib appears to have a safer and a durable disease control in pretreated R/M NPC patients, making it a potential drug to be used in combination with ICIs, chemo, or radiotherapy. In 2020, apatinib (novel VEGFR-2 inhibitor) efficacy was evaluated, reporting an ORR of 31.37%, PFS of 9 months and OS of 16 months [100]. The treatment was well tolerated with the most common adverse events being hypertension and hand-foot syndrome. Axitinib and apatinib appear to be the most tolerable drugs to be potentially used in combination with cytotoxic drugs.

Even though VEGFR inhibitors have not demonstrated an improvement in patient survival and response, the high expression of VEGFR in NPC cells and their anti-cancerous effects indicate that this therapy must continue to be explored. This will offer new drugs to be developed with higher efficacy, and less adverse events.

#### Therapies targeting Epstein-Barr virus

Immunotherapeutic approaches are focused in targeting viral-associated malignancies. The idea under this approach is the simulation of the immune system (mainly T cell) against viral antigens expressed in NPC cells. This immunological therapy is under research in poorly differentiated (WHO type II) and undifferentiated (WHO type III) NPC.

##### Cytotoxic T cells and tumor-infiltrating lymphocytes

Adoptive immunotherapy is a distinctive way to promote immune response avoiding the antigen presentation and direct activation of effector cells. Many preclinical studies have investigated the use of cytotoxic T cells (CTLs) and tumor-infiltrating lymphocytes (TILs) in the treatment of R/M NPC-EBV+. As NPC tumor cells are commonly infected by EBV, the idea of using the virus as a target therapy is possible via de identification of EBV-specific CTL precursors (CTLp) in patients’ blood. CTLp varies between healthy patients and NPC patients, with values being lower in the latter and these changing depending on disease stage [101]. Infused autologous EBV-CTLs in advanced NPC patients demonstrated a regular increase in CTLp levels restoring host surveillance of EBV replication and reducing plasma EBV burden [101].

Phase I trials have demonstrated that autologous CTLs can be used treating advanced NPC. One study maintained 4 patients in remission disease free and 6 patients with refractory disease, 2 obtained complete response and remain in remission for 11–23 months posttreatment [102]. A similar study demonstrated in ten patients with stage IV NPC that progressed to chemotherapy a control of disease progression in 6 patients and induction of specific LMP-2 response against EBV [103]. Gottschalk and Louis et al. showed that EBV-CTLs were safe, have antitumor activity and that CTLs specificity for a particular EBV antigen influences outcome [104]. Additionally, they demonstrated PFS rates at 1 and 2 years were of 65% and 52% respectively with OS rates of 87% and 70%. Another trial treated 21 patients who progressed to first-line chemotherapy and even though the survival rates and response were not as expected (2.2 months PFS and 16.7 months), two positive outcomes were obtained [105]. One patient achieved complete response and had been in remission for >8 years, and two patients who failed EBV-CTLs demonstrated a better response to previously failed chemotherapy regimens. Demonstrating that EBV can be used as a target therapy via CTLs.

Few studies evaluate CTLs as first-line treatment. Chia et al. evaluated its safety after gemcitabine-carboplatin, resulting in an ORR of 71.4%, a 2- and 3-year OS rate of 62.9% and 37.1%, respectively and five patients not requiring additional chemotherapy for 34 months since CTL initiation [106]. These results achieved extremely positive outcomes in R/M NPC patient survival, so much that in 2022 results from a phase III trial (VANCE) were published. The VANCE trial compared gemcitabine-carboplatin followed by EBV-CTL versus gemcitabine-carboplatin alone. OS in the experimental group was 25 months, demonstrating no benefit over standard of care (24.9 months), a shorter PFS (7.9 vs 8.5 months); however, it maintained a safety profile [107]. Regarding treatment response, ORR and CBR were similar for both the experimental and standard chemotherapy group with an ORR of 61.0% and 63.3% and a CBR of 84.8% and 81.9%, respectively. The ongoing NCT03769467 trial analyzing tabelecleucel (allogeneic EBV specific T cell immunotherapy) + pembrolizumab in EBV+ NPCs was terminated in 2022 with no results posted yet. Finally, TILs have been applied in locoregionally advanced NPC postconcurrent chemoradiation therapy. This therapy was studied in 23 patients of which 20 exhibited an ORR, with measurable plasma EBV-DNA not detectable in 17 patients after 6 months of treatment [108]. TIL´s may be effective and safe, with potential use in the management of advanced NPC patients and should be studied further.

These trials demonstrate that targeting EBV via CTLs or TILs is an effective method, with minimal adverse events. The development of new technologies improving these therapies and adequate clinical trials, CTLs, or TILs could become a new standard of care in the future.

##### Vaccines

Vaccines are a promising therapeutic in multiple types of cancer, including NPC, as it is a viral driven neoplasia. Most vaccine trials are phase 1, but with promising results. In 2002, immunization with EBV peptide-pulsed dendritic cells was performed via direct injection into inguinal lymph nodes of NPC patients who recurred. The immunization induced a functional CD8 T cell response against EBV and caused a partial tumor reduction [109].

The goal of vaccines is to stimulate a T cell response against EBV antigens expressed in tumor cells and induce tumor apoptosis. One of the most recognized trials for NPC vaccines (Ankara) used modified gene sequencing of EBV strains and combined the two main EBV antigens EBNA1 and LMP2. This vaccine demonstrated an effective reactivation of CD4 memory T cells specific for EBNA1 and CD8 memory T cells specific for LMP2, which are able to boost the immune system against NPC tumor cells specifically [110]. These findings made Ankara a potential therapeutic strategy that was evaluated in endemic NPC patients. The trial evaluated patients in remission, reporting an increase in T cell response to one or both EBV antigens with the response being directly associated with vaccine dose [111]. Later on, the same approach was performed in non-endemic NPC. This trial also reported an increased immunity to either one or both viral antigens, recognition of epitopes between EBV strains and functional differentiation of T cells specific for EBNA1 and LMP2 [112]. This vaccine shows anti-cancerous effects in both endemic and non-endemic cases.

A different approach was performed by Chia et al., as they immunized patients with autologous dendritic cells transduced with an adenovirus with truncated LMP1/LMP2 proteins. Similar to the Ankara trials, no severe toxicities were reported, activation of specific T cells against LMP1/2 in vitro but no increase of these peripherally [113]. Nevertheless, 3/16 patients had clinical responses making it a safe and immunogenic vaccine. Similar results have been reported in other vaccines also targeting LMP2, elevating IL-2, IF-y, natural killer cells, CD4 T cells and reducing serum EBV-DNA levels after vaccination [114]. More recently at the ASCO 2020 annual meeting, results from the NCT03282617 trial evaluating the CD137L-DC-EBV-VAX were published. This vaccine has the goal of stimulating CD137L-dendritic cells against LMP1/2. Five patients reported clinical benefit, a rise in IFN-y, PFS of 26 weeks, and patients with a neutrophil:lymphocyte ratio under 3 associated with prolonged PFS [115].

All these trials demonstrated to have potent immunogenic activity against EBV antigens, making them potential therapeutic options especially with the goal of maintaining remission or after failed lines. Two clinical trials in which results are expected include NCT01094405 which evaluates a recombinant EBV vaccine (EBNA/LMP2) in patients with residual EBV-DNA load after conventional therapy and NCT01800071 evaluating the immune response of vaccine MVA-EBNA1/LMP2.

##### Chimeric antigen receptor T cell therapy

Chimeric antigen receptor T cell (CAR-T) therapy is a novel therapeutic with increase research in solid tumors due to positive impact managing non-respondent leukemias. The principle of CAR-T therapies is to obtain T-cytotoxic lymphocytes to recognize and eliminate tumor-associated antigens with a high selectivity and destroy neoplastic cells [116]. Its use has demonstrated to be efficacious in cancers that lack or lose EBV antigen expression. CAR-T therapy is an option to fight cancer cells with loss of EBV antigens by targeting other tumor antigens such as CD30 neoplastic cells and destroy EBV-/CD30+ neoplastic cells [117]. The ongoing phase I clinical trial (NCT01818323) evaluates the use of autologous CAR against ErbB (highly expressed in HNC) [118]. Regarding specific effects against EBV+ NPC, Tang et al. demonstrated reduced tumor growth with CAR-T therapy [119]. This study used CAR-T therapy targeting LMP1 NPC cells producing an IL2 and IFN-c response against these cells and reducing tumor growth. CAR-T therapy can be an alternative approach in the management of EBV+ NPCs.

Various trials have been proposed, which include NCT02915445 targeting the epithelial cell adhesion molecule which plays an important role in tumor metastasis and invasion (currently recruiting), NCT02980315 targeting LMP1 via CAR-T (status unknown), NCT04107142 targeting gamma-delta T cells (status unknown), NCT03925896 targeting LMP2 via T cell receptors (TCR) (status unknown), and NCT03648697 targeting LMP1/LMP2/EBNA1 via TCR (status unknown).

##### Oncolytic viruses

This therapy kills cancer cells directly and selectively spreading within the tumor not harming healthy tissue. Oncolytic viruses secrete cytokines and chemokines that facilitate tumor antigen expression which leads to the recruitment of immune cells into the tumors [120]. They may also insert foreign DNA sequences that help in cancer cell selectivity making this therapy safe [121]. One of the major therapeutic advances in oncolytic viruses world for NPC was the approval of H101 (Oncorine) by the Chinese Food and Drug Administration, a genetically modified oncolytic adenovirus, in combination with chemotherapy [122]. This phase III trial demonstrated a 78.8% ORR and a safety profile in favor of H101 + chemotherapy [123].

Other viruses have been studied, such as herpes simplex virus (HSV). A third-generation HSV1 G47Δ demonstrated antitumor effects in EBV+ NPC with complete regression and longer survivals at in vivo models compared to other HSV models [124]. Another HSV oncolytic virus is T-VEC (genetically engineered HSV-Talimogene Laherparepvec) therapy, which alone or in combination with immunotherapy may have potential anti-tumor effects in NPC due to its approval by the FDA for melanoma and promising results HNC [125]. Finally, Smith et al. used an adenoviral vector to transport CTLs, combining a T cell therapy with an oncovirus. This treatment offered an OS of 38.1 months and PFS of 5.5 months making it an alternative therapy in consolidation scenarios post-chemotherapy [126].

Oncolytic virus therapy is a novel approach due to its tumor-specific activity and minimal side effects. Though, more trials are needed, the approval for Oncorine and promising results of G47Δ make oncolytic viruses a therapy that could be use alone or in combination with other therapies.

As observed, novel therapies have been developed for R/M NPC, with anti-cancer effects, promising efficacy, and minimum toxicities. The overall immune model of NPC makes patients fit for immunotherapy, especially ICIs. EBV vaccine, and other adoptive immunotherapy are treatment options for NPC, with promising results to come. Studies involving the molecular and cellular components of immune escape to affect immunotherapy resistance are needed and could lead to new treatment methods improving response and patient outcome. The following table (Table 2) summarizes what we consider to be the most important results from trials involving immunotherapy and targeted molecular therapies for R/M NPC.

**Table 2:** Selected clinical trials for immunotherapy and targeted therapies in the management of R/M NPC

Trial	Disease setting	Therapy studied	n	ORR	PFS	OS
Immune therapies						
Immune checkpoint inhibitors combination						
RATIONALE-309 Zhang L, et al., 2022. [74]	1^st^ line treatment.	Tislelizumab + gemcitabine + cisplatin vs Placebo + gemcitabine + cisplatin	263	N/A	9.4 months vs 7.4 months	Not reached vs 23.0 months
CAPTAIN-1^st^ Yang Y, et al., 2021. [73]	1^st^ line treatment.	Cambrelizumab + gemcitabine + cisplatin vs Placebo + gemcitabine + cisplatin	263	87.3% vs 80.6%	9.7 months vs 6.9 months	Not reached vs 22.6 months
JUPITER-02 Mai HQ, et al., 2021. [72]	1^st^ line treatment.	Toripalimab + gemcitabine + cisplatin vs Placebo + gemcitabine + cisplatin	289	77.4% vs 66.4%	11.7 months vs 8.0 months	1-year: 91.6% vs 87.1% 2-year: 77.8% vs 63.3%
Fang W, et al., 2018. [80]	1^st^ line treatment.	Camrelizumab + gemcitabine	23	91%	6 months: 86.4% 12 months: 61.4%	N/A
Immune checkpoint inhibitors monotherapy						
KEYNOTE-122 Chan ATC, et al., 2021. [76]	Chemotherapy resistant.	Pembrolizumab vs Oncologist´s choice chemotherapy	233	21.4% vs 23.3%	4.1 months vs 5.5 months	17.2 months vs 15.3 months
POLARIS-02 Wang FH, et al., 2021. [79]	Chemotherapy resistant.	Toripalimab	190	20.5%	1.9 months	17.4 months
CAPTAIN Yang Y, et al., 2021. [81]	Chemotherapy resistant.	Camrelizumab	156	28.2%	3.7 months	17.4 months
Even C, et al., 2021. (129)	Chemotherapy resistant.	Spartalizumab vs Oncologist´s choice chemotherapy	122	17.1% vs 35.0%	1.9 months vs 6.6 months	25.2 months vs 15.5 months
AK105-202 Chen X, et al., 2020. (130)	Chemotherapy resistant.	Penpulimab	130	27.0%	3.7 months	18.6 months
Lim D, et al., 2019. [82]	Chemotherapy resistant.	Spartalizumab vs Oncologist´s choice chemotherapy	113	18.4% vs 32.4%	1.9 months vs 6.6 months	N/A
Wang S, et al., 2019. (131)	Chemotherapy resistant.	Tislelizumab	21	43%	4.0 months	1-year: 60%
NCI-9742 Ma BBY, et al., 2018. [77]	Chemotherapy resistant.	Nivolumab	44	20.5%	1-year: 19.3%	1-year: 59%
Fang W, et al., 2018. [80]	Chemotherapy resistant.	Camrelizumab	93	34%	5.6 months	N/A
Shen L, et al., 2018. (132)	Chemotherapy resistant.	Atezolizumab	20	10%	N/A	N/A
KEYNOTE-028 Chiun Hsu, et al., 2017. [43]	Chemotherapy resistant with PD-L1 >1%.	Pembrolizumab	27	25.9%	6 months: 50% 12 months: 33.4%	6 months: 85.2% 12 months: 63.0%
CheckMate-358 Delord JP, et al., 2017. [78]	≤ 2 prior systemic therapies.	Nivolumab	24	20.8%	2.4 months	Not reached
CTLs and TILs						
VANCE Toh HC, et al., 2022. [109]	1^st^ line treatment EBV +.	Carboplatin-gemcitabine + Autologous EBV-specific CTLs vs carboplatin-gemcitabine	330	61.0% vs 63.3%	7.9 months vs 8.5 months	25 months vs 24.9 months
Walsh RJ, et al., 2020. [117]	Chemotherapy resistant.	Autologous CD137L-dendrtici cell with EBV (LMP1/LMP2) vaccine. (CD137L-DC-EBV-VAX)	14	8.3%	26 weeks	N/A
Huang J, et al., 2017. [107]	Chemotherapy resistant EBV +	Autologous EBV-specific CTLs	28	4.8%	2.2 months	16.7 months
Chia WK, et al., 2013. [108]	1^st^ line treatment EBV +.	Cisplatin-gemcitabine + Autologous EBV-specific CTLs	38	71.4%	7.6 months	29.9 months
Secondino S, et al., 2012. (133)	Chemotherapy resistant EBV +.	Autologous EBV-specific CTLs	11	18.18%	N/A	N/A
Louis CU, et al., 2010. [106]	Chemotherapy resistant EBV+	Autologous EBV-specific CTLs	23	48.7%	1-year: 65% 2-years: 52%	1-year: 87% 2-years: 70%
Straathof KCM, et al., 2005. [104]	Remission or chemotherapy resistant EBV+	Autologous EBV-specific CTLs	10	30%	N/A	N/A
Comoli P, et al., 2005. [105]	Chemotherapy resistant EBV+	Autologous EBV-specific CTLs	10	20%	N/A	N/A
Vaccines						
Chia WK, et al., 2012. [115]	Chemotherapy resistant.	Adenovirus vaccine (Ad-LMP1-LMP2)	16	6.25%	1.92 months	6.0 months
Lin CL, et al., 2002. [111]	Chemotherapy resistant.	Autologous dendritic cells vaccine with HLA epitopes LMP2	16	12.5%	N/A	N/A
Oncolytic viruses						
Smith C, et al., 2017. (128)	Active R/M disease and minimal residual disease	Adenoviral vector (AdE1-LMPpoly)-based CTLs	29	0%	5.5 months	38.1 months
Xia ZJ, et al., 2004. [125]	Chemotherapy resistant.	Intratumoral adenovirus vaccine (H101) + cisplatin + 5FU or adriamycin + 5FU vs cisplatin + 5FU or adriamyacin + 5FU	123	78.8% vs 39.6%	N/A	N/A
Targeted therapy						
EGFR inhibitors						
You B, et al., 2012. [11]	Maintenance therapy postchemotherapy	Erlotinib maintenance postgemcitabine-platinum	20	0%	6.9 months	12 months: 80%
Chua DTT, et al., 2008. [91]	Chemotherapy resistant.	Gefitinib	19	0%	4 months	16 months
Ma B, et al., 2008. [92]	Chemotherapy resistant.	Gefitinib	16	0%	2.7 months (mean)	12 months (mean)
Chan ATC, et al., 2005. [54]	Chemotherapy resistant EGFR+.	Cetuximab + carboplatin	60	11.7%	81 days	233 days
VEGFR inhibitors						
Li L, et al., 2020. [102]	Chemotherapy resistant.	Apatinib	51	31.37%	9 months	16 months
Jiang W, et al., 2019. (134)	Chemotherapy resistant.	Apatinib	33	36.3%	5.0 months	1-year: 83.1%
Hui EP, et al., 2018. [101]	Chemotherapy resistant.	Axitinib	40	2.7% (confirmed) 18.9% (unconfirmed)	5.0 months	10.4 months
Jin T, et al., 2018. (135)	1^st^ line treatment.	Endostar + cisplatin + gemcitabine	72	77.8%	12 months	19.5 months
Huang Y, et al., 2013. (136)	Chemotherapy resistant.	Famitinib	58	8.6%	3.2 months	N/A
Jin T, et al., 2013. (137)	1^st^ line treatment.	Endostar + cisplatin + gemcitabine	30	85.7%	19.2 months	1-year: 90.2%
Xue C, et al., 2012. [98]	1^st^ line treatment.	Sorafenib + cisplatin + 5FU	54	77.8%	7.2 months	11.8 months
Lim WT, et al., 2011. [99]	Chemotherapy resistant.	Pazopanib	33	6.1%	4.4 months	10.8 months
Hui EP, et al., 2011. [100]	Chemotherapy resistant.	Sunitinib	13	7.1%	3.5 months	10.5 months
Elser C, et al., 2007. [97]	≤ 1 prior chemotherapy	Sorafenib	27	3.7%	1.8 months	4.2 months

*ORR*, overall response rate; *PFS*, progression free survival; *OS*, overall survival; *N/A*, not available or not reported

## Ongoing phase 2/3 clinical trials

There are multiple ongoing phase 2/3 clinical trials in recurrent/metastatic NPC evaluating different novel therapies for first and second or subsequent lines of therapy. Table 3 summarizes these ongoing trials, if they are being analyzed for EBV + or -, their therapeutic target as well as future therapies and targets that will be seen in trials to come.

**Table 3:** Ongoing phase II/III clinical trials for metastatic or recurrent NPC depending on first or second/more lines of treatment and their EBV viral status

NCT ID	Phase	Viral status	Purpose/primary objective		Targets	Primary outcome
First-line treatment						
NCT03097939 (recruiting)	II	EBV positive	Evaluate the impact in overall response rate that nivolumab + ipilimumab has in EBV + NPC.		PD-1 CTLA-4	Overall Response Rate
NCT04974398 (recruiting)	III	EBV positive	Evaluate the efficacy and safety of penpulimab + cisplatin + gemcitabine as first-line treatment.		PD-1	Progression free survival.
NCT05162872 (recruiting)	II	Not specified	Efficacy and safety of niraparib + sintilimab in first-line or recurrent scenarios.		PARP PD-1	Overall Response Rate
NCT04458909 (active not recruiting)	III	Not specified	Compare efficacy and safety of adding nivolumab to standard chemotherapy.		PD-1	Overall survival
NCT05385926 (recruiting)	II	Not specified	Safety and efficacy of first-line cisplatin-gemcitabine + toripalimab combined with radiotherapy.		PD-1	Progression free survival
Second or more lines of treatment						
NCT03422536 (completed awaiting results)	II	EBV negative	To assess the efficacy of ficlatuzumab, with or without concurrent cetuximab in cetuximab-resistant patients.		c-MET EGFR	Progression free survival
NCT03915678 (recruiting)	II	EBV positive	Assess the effects of atezolizumab + BDB001 + radiotherapy in multiple solid tumors, including viral associated tumors (e.g., NPC EBV +).		PD-L1 TLR-7	Disease control rate.
NCT03357757 (recruiting)	II	EBV positive	Evaluate the feasibility and efficacy of valproic acid combined with avelumab for the treatment of virus associated cancers.		GABAr PD-L1	Clinical response rate.
NCT04825990 (recruiting)	II	EBV positive	Evaluate the efficacy and safety of pembrolizumab + olaparib in platinum resistant scenarios.		PD-L1 PARP	Overall Response Rate
NCT05166577 (recruiting)	II	EBV positive	Evaluate the safety and efficacy of nanatinostat + valganciclovir with or without pembrolizumab.		HDAC DNA pol PD-L1	Overall Response Rate
NCT04396886 (active not recruiting)	II	EBV positive	Evaluate the efficacy and safety of bintrafusp alfa in previously treated non-keratinizing NPC patients.		PD-L1/TGFβ	Objective tumor response
NCT04996758 (recruiting)	II	Not specified	Evaluate efficacy and safety of toripalimab + anlotinib in patients with recurrent or metastatic disease after failure of ≥ 1 platinum-based chemotherapy.		PD-1 TKI (VEGFR, FGFR, PDGFR)	Overall Response Rate and Disease Control Rate
NCT04978012 (recruiting)	II	Not specified	Evaluate the efficacy and safety of fluzoparib + camrelizumab.		PARP PD-1	Overall Response Rate
NCT04872582 (active not recruiting)	II	Not specified	Evaluate the efficacy and safety of PD-1 inhibitors + bevacizumab after failure of first-line chemotherapy.		PD-1 VEGFR	Overall Response Rate
Other agents / combinations with future potential impact						
Drug				Target		
Triprilimab				PD-1		
Camrelizumab				PD-1		
Surufatinib				VEGFR, FGFR1, CSF-1R		
Apatinib				VEGFR		
Bevacizumab + cetuximab				VEGFR EGFR		
Camrelizumab + apatinib				PD-1, VEGFR		
Triprilimab + surufatinib				VEGFR, FGFR1, CSF-1R		

## Conclusion

R/M NPC generally has a poor prognosis; currently, we know platinum-doublet chemotherapy is a good option. Ongoing research in this scenario have had promising results and improved patient survival. The advances in the area of immunotherapy combined with chemotherapy or as monotherapy have improved outcome and survival. Other immunological therapies and molecular targeted therapies are currently being under investigation and may allow oncologists to have additional options for this disease. Extensive full exome sequencing has not demonstrated any actionable signatures for this entity. Ongoing research is needed in viral and epigenetic pathways.
